# Dietary patterns and physical activity in young South Asians and white Europeans and their potential implications for cardiovascular risk

**DOI:** 10.1038/s41598-025-97605-z

**Published:** 2025-04-15

**Authors:** Sophie Richardson, Janice Marshall, Catarina Rendeiro

**Affiliations:** 1 School of Biomedical Sciences, College of Medical and Dental Sciences, University of Birmingham, Edgbaston, Birmingham, B15 2TT UK; 2 School of Sport, Exercise and Rehabilitation Sciences, University of Birmingham, Birmingham, B15 2TT UK; 3 Centre for Human Brain Health, University of Birmingham, Birmingham, UK

**Keywords:** Ethnicity, Physical activity, Diet, Cardiovascular disease, Flavonoids, Cardiovascular diseases, Risk factors

## Abstract

Individuals of South Asian (SA) ethnicity have greater risk of developing cardiovascular disease (CVD) relative to white Europeans (WEs). Factors which generally contribute to increased CVD risk include physical inactivity and poor dietary habits, including high intake of salt and saturated fat. Contrastingly, diets rich in fibre, antioxidants and polyphenols are considered cardioprotective. The current questionnaire-based study aimed to examine whether the dietary habits and physical activity levels of young adult SAs living in the UK may contribute to their increased CVD risk in comparison to age-matched WEs. All participants (80 healthy individuals, 40 SA/ 40 WE (gender-balanced, aged 18–26 years) completed questionnaires to assess: general health; habitual physical activity levels, assessed by the International Physical Activity Questionnaire; and dietary patterns, assessed by EPIC-food frequency questionnaire and three-day food diaries. SAs had higher sitting times (SA: 469 ± 19.4, WE: 387 ± 21.5 min/day, *p* = 0.0107) and were less physically active (SA: 2050 ± 1110, WE: 4850 ± 2810 MET mins/day, *p* < 0.0001) than WEs. Further, SAs had lower consumption of cardioprotective nutrients, such as fibre (*p* = 0.0183), folate (*p* = 0.0242), vitamin C (*p* = 0.0105) and phytochemicals, such as flavonoids (*p* = 0.0644). SAs also consumed less alcohol (*p* < 0.0001), fat (*p* = 0.0066), sugar (*p* = 0.0218) and sodium (*p* = 0.0011) compared to WEs. These findings suggest that lower consumption of nutrients and phytochemicals that are cardioprotective, rather than excess consumption of fat, sugar and sodium, amongst young SAs may contribute to their increased CVD risk. Young SA individuals may also reduce their future CVD risk by increasing their physical activity.

## Introduction

South Asian (SA) ethnicity is a major risk factor in western countries for the development of cardiovascular disease (CVD), with incidence of CVD-associated death estimated to be two- to three- times higher in SAs compared to the wider population^1–3^. Studies also indicate higher prevalence of CVD risk factors, such as hypertension, in SAs compared to White Europeans (WE)^4,5^. Whilst there is some evidence supporting the hereditary nature of CVD, particularly in SAs^6^, lifestyle factors such as smoking, physical activity (PA) and diet are also shown to contribute heavily towards the risk of CVD^7^. Thus, there is most likely an interaction between genetics and lifestyle in determining CV health^8,9^.

Studies suggest that there is a dose-response relationship between PA levels and CVD-risk, with high leisure PA reducing CVD-risk by 20–30%, and moderate PA reducing risk by 10–20% compared to low activity levels^10^. Furthermore, there is evidence of a non-linear association between sedentary time and CVD incidence^11^: higher sitting time has been shown to increase risk of CVD, type-2 diabetes and hypertension, even when adjusting for PA levels^12,13^. It is therefore recommended that adults avoid sedentary behaviour by partaking in at least 150 min of moderate-intensity activity per week^14^.

Importantly, there is evidence of ethnic differences in physical activity; SA culture is commonly associated with low levels of PA, even amongst those living in Western countries, and studies suggest that at least 40% of SAs do not meet recommended activity levels^15,16^. For example, physical inactivity has been shown to be approximately 20% higher amongst SAs^17^ than WEs, and SA total activity levels have been reported to be 31% lower than WEs^18^. Ethnic differences in activity levels are also apparent in children in the UK, with SAs found to have lower daily step counts than WEs and poorer fitness, which is at least partly attributable to reduced PA^19,20^. This suggests that from a young age, SAs are not as exposed to PA, and that continues into their adult life. However, studies formally comparing PA in young healthy SA and WEs adults are limited. Indeed, most published studies focus on older adults or populations of mixed demographics^15–18^.

Diet is another key modifiable risk factor for prevention of CVD^21^. For example, diets such as the Mediterranean diet^22,23^ and DASH diet^24,25^ are widely considered protective against CVD. Commonalities of these healthy dietary patterns include high intake of fibre, antioxidants, vitamins and polyphenols, combined with reduced intake of salt, saturated fats and refined sugars^26^. Specifically, CVD risk is strongly and inversely associated with fruit and vegetable consumption; a 4% reduction in cardiovascular-related mortality has been observed for each additional daily serving of fruit and vegetables^27,28^. Furthermore, consumption of fruits and vegetables rich in flavonoids (a subgroup of polyphenols) can be particularly beneficial for CV health, including reducing risk of hypertension^29^ and reducing CVD incidence and mortality^30,31^. A comprehensive dose-response meta-analysis showed that consumption of 500 mg of daily flavonoids was associated with 27% lower CVD risk^30^. In particular, dietary polyphenols have been shown to improve endothelial function and modulate oxidative stress, which are likely key protective mechanisms against CVD^32^.

Studies directly comparing dietary patterns between WE and SA populations are very limited, with no studies quantifying intake of flavonoids in SA populations. However, a recent review which included adolescents through to those of older age, suggested that SA immigrants have lower consumption of protein and monosaturated fat, as well as micronutrients such as potassium, sodium, and vitamin A than Western populations, but with mixed findings for other nutrients^33^. The SA diet is also traditionally high in saturated fats, such as ghee and cooking oil, and migration to Western countries can lead to further increases in fat and energy consumption due to the addition of other Western high-fat food items^34,35^. SAs also have some of the lowest consumption of fruit and vegetables worldwide, with studies suggesting that fewer than 4% of SAs consume the recommended 400 g/day of fruit and vegetables^36^. It is therefore very likely that dietary choices may contribute to increased CVD risk amongst SAs.

Identifying modifiable risk factors (such as diet and physical activity) within a young healthy population is particularly critical, as behavioural changes around PA and diet, are likely to help mitigate CVD risk and future health outcomes. To our knowledge, no studies have directly quantified and compared dietary patterns, PA and sedentary time in young healthy WE and SA adults. Furthermore, specific key aspects of diet, such as intake of flavonoids, now known to be important contributors to cardiovascular health, have never been quantified and compared across these two ethnic groups. Thus, the present study aimed to directly compare dietary intake (including macronutrients, targeted micronutrients, and phytochemicals, such as flavonoids), PA and sedentary time within and between young men and women of SA and WE ethnicity.

## Methods

### Participants

We recruited a total of 80 men and women (*n* = 40 WE and *n* = 40 SA, aged 18–26 years) via email and social media advertisements, from February 2021 to September 2022. Participant ethnicity was self-reported on the basis of several factors including common ancestry and elements of culture, identity, religion, language and physical appearance, in accordance with guidelines from the Office of National Statistics^37^. Participants were required to have both parents of the same ethnic origin, and other inclusion criteria were that they must be English-speaking UK residents, and aged 18–26 years at the time of the study. Each participant signed a consent form committing to the study and the use of their data as outlined in the Participant Information Sheet, in accordance with approval granted by the University of Birmingham STEM Ethics Committee (ERN17_1755). Each volunteer was assigned a random four-digit ID number for use throughout the study to ensure anonymisation.

### Data collection

The survey was conducted using SmartSurvey^38^. The first component consisted of a general health and lifestyle questionnaire (including questions relating to family heritage and health); the EPIC food frequency questionnaire (FFQ), which is widely used to assess participants’ average weekly consumption of various foods during the last year in order to estimate their nutrient intake^39^; and International Physical Activity Questionnaire (IPAQ), which relates to participants’ activity levels during a seven-day period and responses are used to quantify their physical activity at vigorous to light intensities, as well as overall^40^.

Upon completion of this initial questionnaire participants were then sent a link for the three-day food diary, which they were asked to complete for two weekdays and one weekend day, and reminders to complete this were sent each morning. The food-diary was used alongside the FFQ to provide a more detailed insight into participants’ daily diet and to provide an alternative means of calculating nutrient intake over a shorter time-period. Participants were asked to eat and drink as they normally would, and to record everything they consumed throughout the day. Instructions, and examples of detail, were provided, and participants were instructed to provide quantities, brand names and cooking methods, as well as nutrient supplement usage and the option to include recipes for home-cooked meals. Following the first day of data collection, participant’s food diaries were checked for accuracy and feedback was given as necessary. Unfortunately, the uptake for this part of the survey was lower than for the initial questionnaire and some responses had to be excluded due to insufficient detail for accurate analysis.

### Data analysis: physical activity and habitual dietary intake

Results from the IPAQ were interpreted manually using Excel. Energy expenditure was estimated by multiplying the metabolic equivalent of on activity (MET) by the minutes spent doing it to give measures in MET mins for exercise at light, moderate and vigorous intensities^41^.

The FFQ data was interpreted using FETA software, as previously reported^39^. In order to calculate flavonoid intake, the FLAVIOLA food composition database was inputted into the FETA software, which allows the estimation of total flavonoids and subclasses (flavanols, flavonols, flavones, flavanones, anthocyanins)^42^. Intake of fruit and vegetables estimated from the FFQ was used to calculate daily portions, with one portion corresponding to 80 g.

Food-diary responses were manually processed into a dietary software analysis package, Dietplan 7.0 (Forestfield Software Ltd., Horsham, West Sussex, UK), which utilises UK-specific databases such as McCance and Widdowson, as well as relevant ethnic minority food databases. The USDA database was used for assessment of polyphenol (flavonoid) intake. Food items which could not be found in the software databases were added using food manufacturing websites and composition tables. All nutrients were then exported to calculate daily intake, with relevant nutrients selected for data analysis.

In order to validate dietary intakes estimated from the FFQ and food diary, energy intakes (kcal) were compared against individual energy requirements. These were estimated using the Goldberg equation; individuals’ basal metabolic rates were calculated from their BMI (using age- and gender-specific equations) and this was multiplied by an index of physical activity level^43^. A standard value of 1.55 was applied for physical activity, which represents the WHO recommended minimum activity level^44^. Though this may be a conservative estimate of physical activity in some participants in this study, it minimises the potential influence of misreported activity levels from the self-reporting questionnaire used and is recommended to provide reasonable sensitivity and specificity for the sample size and dietary assessment tools used^43,45^. A ratio of energy intake to expenditure between 0.76 and 1.24 was considered ‘acceptable’, values less than 0.76 or greater than 1.24 were indicative of ‘under’ and ‘over’ reporters respectively, as reported in previous studies^45^.

### Statistical analysis

All statistical analysis was performed and figures were created using Graphpad Prism Version 9.2.0. Anthropometric measures, IPAQ, FFQ and food diary results were analysed using two-way analysis of variance (ANOVA) for main effects of ethnicity and/or gender, followed by post-hoc Tukey’s pairwise comparisons where an ethnicity x gender interaction was identified. Smoking habits, parental health, body mass index (BMI) and student status were compared by Fisher’s exact test. Pearson’s correlation coefficients were used to assess the relationship between FFQ and food diary data; these were calculated by converting the intake of all key macronutrients that had been assessed by both methods into gram measures, and combining these in the correlation analysis. Outliers (classified as greater than two standard deviations from the mean of each group) were identified prior to group comparisons. One outlier was excluded from anthropometric measures and a total of ten were excluded from IPAQ calculations (at all intensities). For the FFQ and food-diary, outliers were calculated independently for each nutrient. If the same participant was an outlier for three or more nutrients, they were excluded across all analysis for that questionnaire. However, for individuals who were only outliers in a single nutrient, only the relevant data points were excluded. A significance level of *p* < 0.05 was used for all statistical analyses, and all values reported are mean ± SD.

## Results

### Population characteristics

Anthropometric measures are shown in (Table 1), *n* = 39 WE (20 men, 19 women) and *n* = 40 SA (20 men, 20 women). WEs were significantly older [F(1,75) = 5.46, *p* = 0.0221], taller [F(1,75) = 11.3, *p* = 0.0012], and had a lower BMI [F(1,75) = 10.4, *p* = 0.0019] than SAs, but no effect of ethnicity on weight [F(1,75) = 0.925, *p* = 0.339]. There was also a significant ethnicity x gender interaction for BMI [F(1,75) = 6.12, *p* = 0.0156]; post-hoc tests highlighted that SAs had a higher BMI than WEs only in women (*p* = 0.0008). According to NHS guidelines for healthy BMI levels (WEs: <25 kg/m^2^, SA: <23 kg/m^2^), significantly more WEs (72.5%) were within the healthy range compared to SAs (35%, *p* = 0.0015). Furthermore, though only one WE participant was classified as obese (> 30 kg/m^2^), significantly more SAs were above the threshold BMI for SAs to be classified as obese (27.5%, *p* = 0.0033).

**Table 1:** Anthropometric data presented for groups of white European (WE) and South Asian (SA) men (M) and women (F). Data shown is mean ± SD, and ethnic differences from 2-way ANOVA (*p < 0.05, **p < 0.01).

		White European (WE)	South Asian (SA)
		*n* = 39, M:20, F:19	*n* = 40, M:20, F:20
Age (yrs)	**Overall**	**23.2 ± 1.56**	22.1 ± 2.20*
	F	23.5 ± 1.54	22.3 ± 2.31
	M	22.9 ± 1.56	22.0 ± 2.13
Height (cm)	**Overall**	**174 ± 10.2**	169 ± 10.7**
	F	167 ± 6.10	160 ± 4.79
	M	181 ± 8.48	178 ± 6.53
Weight (kg)	**Overall**	**70.8 ± 13.4**	**72.3 ± 13.5**
	F	61.5 ± 9.40	65.6 ± 13.8
	M	80.0 ± 10.1	78.9 ± 9.50
BMI (kg/m^2^)	**Overall**	**23.2 ± 3.16**	25.2 ± 3.63**
	F	22.1 ± 3.33	25.5 ± 4.36
	M	24.4 ± 2.56	24.9 ± 2.79

There was no difference between the proportion of non-smokers in WE and SA populations (WE: 69.2%, SA: 75.0%, *p* = 0.622). However, men were significantly more likely to be smokers compared to women (men: 43.6%, women: 12.5%, *p* = 0.0026).

The prevalence of parental cardiovascular conditions (hypertension, type 2 diabetes, high cholesterol) was higher in SAs than in WEs: 62.5% of SAs reported at least one parent with a cardiovascular condition compared with 20.0% of WEs (*p* = 0.0002). There was no gender difference in parental cardiovascular health (*p* > 0.999). Hypertension was the most commonly reported condition in both ethnic groups; the prevalence of parental hypertension was significantly higher in SAs than WEs (SA: 40.0%, WE: 15.0%, *p* = 0.0230). There was no difference in the prevalence of parental hypertension between men and women (M: 22.5%, F: 32.5%, *p* = 0.453).

All except two WE participants were born in the UK, and all were born in Europe. Contrarily, 50.0% of SA participants were born outside of Europe (10 men and 10 women). Within this group, 25.0% have lived in the UK for over ten years, 20.0% for six to ten years, and 25.0% for five years or less (30.0% of respondents did not indicate when they moved to the UK).

75.0% of WE and 65.0% of SA participants were students, the remaining participants were in a mixture of full- and part-time employment in the workplace and working from home. The group sizes were too small for further analysis on this basis.

### Habitual physical activity and sitting time

**Fig. 1:**
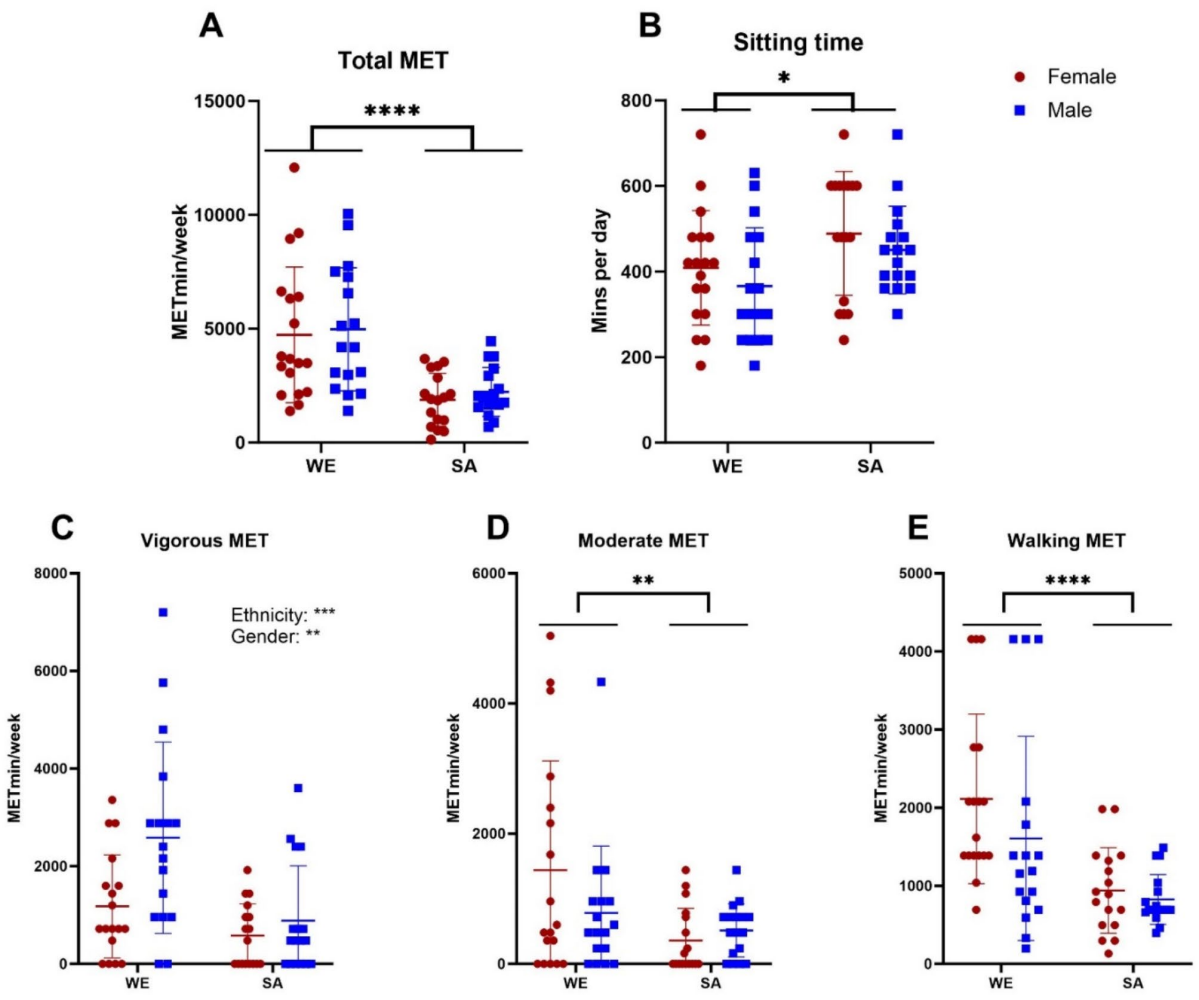
Distribution of physical activity within groups of white European (WE) and South Asian (SA) women (red) and men (blue), as derived from the International Physical Activity Questionnaire for (**A**) total, (**B**) vigorous, (**C**) moderate and (**D**) walking activity. Data presented are mean ± SD alongside p values for main effects of gender and/or ethnicity from 2-way ANOVA (*p < 0.05, **p < 0.01, ***P < 0.001, ****p < 0.0001).

WEs had significantly higher total MET min/week than SAs [WE: 4850 ± 2810; SA: 2050 ± 1110; F(1,65) = 28.5, *p* < 0.0001], but with no main effect of gender on total MET min/week [F(1,65) = 0.317, *p* = 0.576, Fig. 1A]. Daily sitting time, assessed by the IPAQ, was significantly higher in SAs (469 ± 19.4 min) than WEs [387 ± 21.5 min, F(1,65) = 6.92, *p* = 0.0107, Fig. 1B]. There was no significant difference in sitting time between men (408 ± 21.7 min) and women [447 ± 24.2 min, F(1,65) = 1.70, *p* = 0.197].

WEs had significantly higher MET min/week than SAs across all intensities, as shown in (Fig. 1C–E) [Vigorous- WE:1880 ± 1700, SA:732 ± 916, F(1,65) = 13.8, *p* = 0.0004, moderate- WE:1120 ± 1420, SA:436 ± 454, F(1,65) = 7.16, *p* = 0.0094, walking- WE:1870 ± 1210, SA: 883 ± 445, F(1,65) = 19.8, *p* < 0.0001]. At vigorous intensity only, men were also more active than women [F(1,65) = 7.64, *p* = 0.0074, Fig. 1C].

### Habitual dietary intake

Mean weekly alcohol consumption was significantly higher in WE (12.1 ± 2.60 units) than SA (2.43 ± 0.875 units) groups [F(1,76) = 54.8, *p* < 0.0001, Supplementary Fig. 1A]. Men (8.98 ± 1.32 units) also had significantly higher alcohol consumption than women (5.50 ± 1.06 units, F(1,76) = 7.14, *p* = 0.0092).

38.0% of participants regularly consumed dietary supplements; those most commonly reported were multivitamins, vitamin C, vitamin D, iron and fish oil. There was no difference in supplement consumption between WEs (35.9%) and SAs (32.5%, *p* = 0.815), but women (48.7%) had significantly more than men (22.5%, *p* = 0.0193).

Daily portions of fruit and vegetables were estimated for each participant from the FFQ; there was no main effect of ethnicity [WE: 5.37 ± 2.54, SA: 5.74 ± 2.60, F(1,65) = 0.396, *p* = 0.531] or gender [F(1,65) = 0.0273, *p* = 0.869] on total daily portions (Supplementary Fig. 1B). There was a significant ethnicity x gender interaction [F(1,65) = 4.02, *p* = 0.0492]; multiple comparisons tests found no significant differences between individual groups. Considering the proportion of the population who meet the recommended daily intake of 5 portions per day, there was no significant difference between WEs and SAs meeting daily recommendations (WE: 48.6%, SA: 51.4%, *p* = 0.811), or between males and females (females: 48.6%, males: 51.4%, *p* = 0.811).

In terms of nutrient intake estimated from the FFQ (Table 2), WEs (2580 ± 955 mg) consumed significantly more sodium than SAs [2060 ± 769 mg, F(1,67) = 7.086, *p* = 0.0097]. WEs also tended to consume more dietary vitamin D than SAs, although this did not reach statistical significance [WE: 2.72 ± 1.83, SA: 2.04 ± 1.28mcg, F(1,65) = 3.58, *p* = 0.063]. There was no ethnic difference in any other nutrients.

Males consumed significantly more energy [F(1,67) = 12.0, *p* = 0.0009], protein [F(1,67) = 12.0, *p* = 0.0009], carbohydrates [F(1,67) = 11.0, *p* = 0.0015], sugar [F(1,66) = 7.06, *p* = 0.0099], fat [F(1,67) = 6.26, *p* = 0.0148], saturated fat [F(1,66) = 9.86, *p* = 0.0025], sodium [F(1,67) = 8.30, *p* = 0.0053], cholesterol [F(1,67) = 7.13, *p* = 0.0095], folate [F(1,67) = 4.06, *p* = 0.0481] and vitamin D [F(1,65) = 4.36, *p* = 0.0408] than women.

**Fig. 2:**
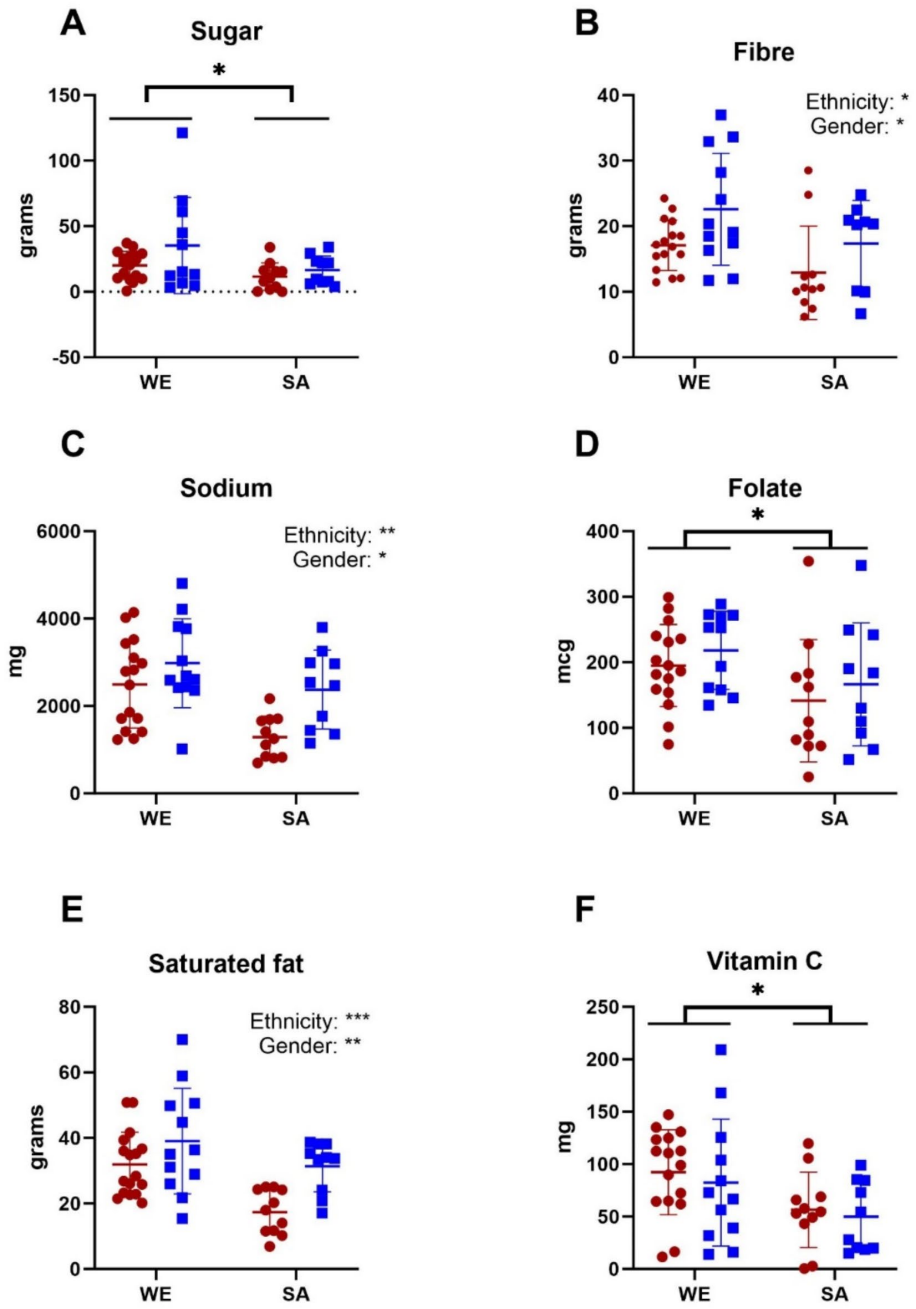
Daily intake of some key nutrients associated with cardiovascular disease risk for white European (WE) and South Asian (SA) women (red) and men (blue), as derived from the three-day food diary. Data shown are mean ± SD alongside main effects of gender and/or ethnicity are presented from 2-way ANOVA (*p < 0.05, **p < 0.01, ***P < 0.001).

The three-day food diary provided a more detailed insight into diet over a three-day period (two weekdays and one weekend day). WEs were found to have higher daily intake of energy [F(1,46) = 9.61, *p* = 0.0033], protein [F(1,44) = 7.26, *p* = 0.0100], carbohydrates [F(1,46) = 8.08, *p* = 0.0066], sugar [F(1,45) = 5.65, *p* = 0.0218], fat [F(1,45) = 8.11, *p* = 0.0066], saturated fat [F(1,46) = 12.7, *p* = 0.0009], fibre [F(1,44) = 6.00, 0.0183], sodium [F(1,46) = 12.1, *p* = 0.0011], vitamin C [F(1,45) = 7.13, *p* = 0.0105] and folate [F(1,44) = 5.45, *p* = 0.0242] than SAs (Table 2; Fig. 2). Meanwhile, men consumed significantly more energy [F(1,46) = 21.5, *p* < 0.0001], protein [F(1,44) = 21.4, *p* < 0.0001], carbohydrates [F(1,46) = 10.3, *p* = 0.0025], fat [F(1,45) = 19.5, *p* < 0.0001], saturated fat [F(1,46) = 11.4, *p* = 0.0015], fibre [F(1,44) = 6.70, *p* = 0.0130] and sodium [F(1,46) = 5.86, *p* = 0.0195] than women. Within SAs, men also consumed significantly more fat than women (*p* = 0.0003), but there were no other gender differences in nutrient intake within either ethnic group.

**Table 2:** Habitual nutrient intake, estimated from the food frequency questionnaire, for white European (WE) and South Asian (SA) men (M) and women (F). Data presented are mean ± SD9, P values from 2-way ANOVA for main effect ethnicity within each technique (*p < 0.05, **p < 0.01, ***p < 0.001).

		FFQ		Food diary	
		White European (WE)	South Asian (SA)	White European (WE)	South Asian (SA)
		*n* = 35,* M:17*,* F:18*	*n* = 36,* M:18*,* F:18*	*n* = 29,* M:12*,* F:17*	*n* = 21,* M:10*,* F:11*
Energy (kcal)	**Overall**	**1770 ± 522**	**1670 ± 575**	**2270 ± 757**	1720 ± 769**
	F	1620 ± 391	1410 ± 557	2020 ± 618	1190 ± 391
	M	1940 ± 599	1920 ± 480	2620 ± 823	2310 ± 651
Protein (g)	**Overall**	**80.5 ± 30.7**	**72.9 ± 23.5**	**88.5 ± 36.6**	68.1 ± 25.4**
	F	70.7 ± 23.8	62.1 ± 19.0	71.9 ± 18.6	52.3 ± 16.1
	M	90.9 ± 34.3	83.8 ± 22.9	111 ± 43.3	87.3 ± 21.3
Carbohydrates (g)	**Overall**	**197 ± 59.9**	**201 ± 68.7**	**84.8 ± 57.4**	49.0 ± 39.2**
	F	181 ± 44.3	171 ± 68.5	68.9 ± 35.2	26.4 ± 19.1
	M	215 ± 70.1	232 ± 55.5	104 ± 19.6	73.9 ± 41.1
Sugar (g)	**Overall**	**93.3 ± 35.0**	**93.6 ± 38.3**	**26.1 ± 25.0**	13.9 ± 10.6*
	F	87.3 ± 26.9	77.1 ± 30.3	20.1 ± 10.6	11.6 ± 10.4
	M	99.7 ± 41.8	109 ± 39.2	35.3 ± 36.7	16.5 ± 10.8
Fat (g)	**Overall**	**71.5 ± 24.6**	**67.4 ± 29.2**	**95.3 ± 29.8**	73.3 ± 34.2**
	F	66.5 ± 19.0	57.0 ± 28.5	88.5 ± 20.0	48.5 ± 15.3
	M	76.8 ± 29.1	77.8 ± 26.7	104 ± 38.6	101 ± 27.8
Saturated fat (g)	**Overall**	**27.2 ± 10.3**	**24.9 ± 12.1**	**34.9 ± 13.0**	24.0 ± 10.1***
	F	24.6 ± 7.78	19.5 ± 9.30	31.9 ± 9.76	17.4 ± 6.77
	M	29.9 ± 12.0	30.0 ± 12.4	39.1 ± 16.1	31.3 ± 7.78
Fibre (g)	**Overall**	**14.7 ± 5.18**	**14.7 ± 4.59**	**19.4 ± 6.75**	14.9 ± 7.06*
	F	15.4 ± 5.36	13.5 ± 4.88	17.1 ± 3.84	12.9 ± 7.11
	M	13.9 ± 5.03	15.9 ± 4.05	22.6 ± 8.53	17.4 ± 6.56
Sodium (mg)	**Overall**	**2580 ± 955**	2060 ± 769**	**2700 ± 1020**	1810 ± 888**
	F	2340 ± 538	1740 ± 694	2490 ± 997	1290 ± 477
	M	2830 ± 1220	2380 ± 720	2980 ± 1020	2370 ± 902
Vitamin C (mg)	**Overall**	**101 ± 46.7**	**106 ± 49.5**	**88.1 ± 49.3**	53.3 ± 33.9*
	F	106 ± 51.1	85.2 ± 40.8	92.4 ± 40.6	56.5 ± 35.9
	M	94.9 ± 42.3	127 ± 49.7	82.4 ± 60.4	49.8 ± 33.1
Vitamin D (mcg)	**Overall**	**2.72 ± 1.83**	**2.04 ± 1.28**	**2.35 ± 1.74**	**2.40 ± 1.79**
	F	2.44 ± 1.65	1.54 ± 0.803	2.50 ± 1.83	2.40 ± 1.51
	M	3.02 ± 2.02	2.52 ± 1.48	2.12 ± 1.65	2.40 ± 2.17
Folate (mcg)	**Overall**	**253 ± 87.0**	**240 ± 64.9**	**204 ± 61.3**	153 ± 92.0*
	F	246 ± 89.7	212 ± 64.8	195 ± 62.6	141 ± 93.1
	M	261 ± 86.0	268 ± 53.0	218 ± 59.6	166 ± 93.9

#### Flavonoid intake

According to flavonoid intake estimated from the FFQ, there was no main effect of ethnicity on total flavonoid consumption, nor on any subgroup of flavonoids (Table 3). Men consumed significantly more flavanones than women [F(1,64) = 5.38, *p* = 0.0236] but there were was no significant gender difference for any other subgroup.

By contrast, daily flavonoid intake estimated from the three-day food diary showed that WEs tended to have higher total flavonoid consumption than SAs [F(1,45) = 3.60, *p* = 0.0644]. When broken down into the flavonoid subgroups, it appears that this is mainly driven by the fact that WEs consumed more flavones [F(1,44) = 4.83, *p* = 0.0334] and flavonols [F(1,46) = 16.5, *p* = 0.0002], and tended to consume more flavanols [F(1,46) = 3.19, *p* = 0.0807] than SAs (Table 3). The only significant gender difference was for anthocyanin intake, for which women consumed significantly more than men [F(1,44) = 4.40, *p* = 0.0417].

**Table 3:** Estimated daily flavonoid intake (by subclass and total) estimated from FFQ and 3-day food diary for groups of white European (WE) and South Asian (SA) men (M) and women (F) data presented are mean ± SD, P values from 2-way ANOVA for main effect ethnicity within each technique (*p < 0.05, **p < 0.01).

		FFQ		Food diary	
		White European (WE)	South Asian (SA)	White European (WE)	South Asian (SA)
		*n* = 35, *M:17*,* F:18*	*n* = 36, *M:18*,* F:18*	*n* = 29,* M:12*,* F:17*	*n* = 21,* M:10*,* F:11*
Total flavonoids (mg)	**Overall**	**269 ± 172**	**284 ± 164**	**179 ± 218**	**75.7 ± 111**
	F	307 ± 189	297 ± 213	204 ± 261	99.2 ± 147
	M	235 ± 152	273 ± 104	146 ± 148	49.9 ± 46.4
Anthocyanins (mg)	**Overall**	**12.1 ± 5.85**	**13.1 ± 7.71**	**11.3 ± 11.7**	**8.15 ± 9.21**
	F	11.9 ± 6.13	10.5 ± 7.05	13.7 ± 13.7	11.1 ± 11.2
	M	12.3 ± 5.71	15.5 ± 7.69	7.78 ± 6.87	4.18 ± 2.76
Flavanols (mg)	**Overall**	**219 ± 170**	**228 ± 156**	**161 ± 244**	**50.8 ± 109**
	F	259 ± 175	251 ± 205	201 ± 292	74.1 ± 145
	M	177 ± 159	206 ± 88.9	104 ± 148	25.2 ± 40.1
Flavanones (mg)	**Overall**	**13.5 ± 13.0**	**17.2 ± 12.9**	**5.34 ± 6.46**	**3.29 ± 6.64**
	F	14.4 ± 14.4	9.67 ± 7.87	6.06 ± 6.94	1.08 ± 3.41
	M	12.7 ± 13.6	24.8 ± 12.7	4.22 ± 5.78	5.74 ± 8.56
Flavones (mg)	**Overall**	**3.17 ± 2.59**	**2.48 ± 2.03**	**0.585 ± 0.381**	0.294 ± 0.480*
	F	2.75 ± 2.31	2.90 ± 2.35	0.625 ± 0.372	0.276 ± 0.222
	M	3.58 ± 2.85	2.02 ± 1.53	0.525 ± 0.406	0.312 ± 0.660
Flavonols (mg)	**Overall**	**18.4 ± 7.06**	**19.3 ± 7.18**	**24.0 ± 16.4**	7.82 ± 8.17**
	F	18.5 ± 8.63	18.3 ± 7.93	25.8 ± 20.2	10.6 ± 9.78
	M	18.4 ± 5.35	20.2 ± 6.49	21.5 ± 8.65	4.78 ± 4.72

#### Comparison between FFQ and food diary

Pearson’s correlation coefficients showed a significant positive correlation between FFQ and food-diary responses across the entire population [*n* = 548, *r* = 0.629 (CI 0.575 to 0.677), *p* < 0.0001], as well as within WEs [*n* = 312, *r* = 0.682 (CI 0.618 to 0.737), *p* < 0.0001] and SAs [*n* = 236, *r* = 0.550, (CI 0.454 to 0.633), *p* < 0.0001].

Considering the validation of dietary intakes estimated from the FFQ in comparison to energy expenditure, the overall mean ratio of energy intake to expenditure was 0.736 ± 0.262. For WEs this was 0.771 ± 0.258 (of which 45% were within the ‘acceptable’ range, and the remainder ‘under-reporting’) and the ratio for SAs was 0.700 ± 0.264 (40% ‘acceptable’, 5% ‘over-reporting’ and the rest ‘under-reporting’).

For the food diary the overall ratio of energy intake to expenditure was 0.836 ± 0.288. This was 0.937 ± 0.274 for WEs (of which 53.8% were within the ‘acceptable’ range, 7.69% were ‘over-reporting’ and ’38.5% were ‘under-reporting’), and 0.700 ± 0.253 for SAs, (58.3% ‘acceptable’ and the rest ‘under-reporting’).

## Discussion

The present observational study in young adult SA and WE men and women found that young SAs were less physically active and had higher sitting times than WEs. The food diaries indicated many ethnic differences in dietary intake, with SAs found to have lower consumption of cardioprotective nutrients, such as fibre, folate and vitamin C and also lower intake of flavonoids, known to improve vascular health. Conversely, SAs also consumed less alcohol, fat, sugar and sodium, which have been associated with poorer vascular and metabolic health outcomes. We propose that SA’s increased risk of CVD may be associated with lower physical activity levels that are present in young adulthood, together with lower consumption of protective nutrients/phytochemicals, rather than with increased intake of dietary components associated with poorer vascular health.

### Population characteristics

There were significant differences in height and BMI, between the ethnic groups, largely driven by SA women having higher BMI than WE women (25.5 ± 4.36 kg/m^2^ and 22.1 ± 3.33 kg/m^2^ respectively). Specifically, the NHS guidelines for a healthy BMI differ between ethnic groups according to CVD risk; for WEs a BMI of < 25 kg/m^2^ is considered ‘healthy’, whereas for Asian and other ethnic groups the threshold for ‘healthy’ weight are 23 kg/m^2^^46^ as such SAs were also more likely to be above the ‘healthy’ BMI range. The higher prevalence of parental hypertension and CVD amongst the young SAs is in line with many previous studies demonstrating the prevalence of such conditions in the SA population as a whole relative to the WE population^4,5,47^. The similarity in proportion of students within each ethnic group also suggests that ethnic differences in lifestyle choices are not attributable to student versus employed lifestyles. Taken together, these observations suggest that the group sampled is representative of typical young WE and SA adults in the UK.

### Physical activity and sitting time

The ethnic differences in PA in the present study are consistent with findings from previous self-reporting questionnaires in large population studies of older participants, indicating that SAs are less physically active than WEs in the UK^17,48^. In line with this, lower MET scores for SAs across all intensities were also demonstrated by Afaq et al. in participants aged 35–85 years, using more objective accelerometer-based data to assess activity levels^18^. The mean reported sitting time for SAs in the present study was 469 ± 19.4 min per day, which aligns with other values from self-reported questionnaires (e.g. 416 min per day^49^) and accelerometers (482–587 min per day^50^). Similarly, the observed sitting time for WEs was 387 min per day, which corresponds closely to published data for students in Europe (397 min per day^51^), .

Both WEs and SAs were comfortably over the American Heart Association Guidelines for adults of 600 METmin/week to confer some protection against CVD^52^. However, there is evidence of a dose-response relationship with further benefits to CV-health obtained with higher levels of PA such that each additional MET-hour of activity per week corresponds to a 1% reduction in CVD-risk^53–55^. In the context of the present study, that would translate into a 46% reduction in CVD-risk in young WEs versus young SAs. Further, various studies have observed differences in CVD incidence, mortality and endothelial function linked to sitting times ranging from four to ten hours per day^56–58^. Meta-analyses indicate associations between increasing sedentary time and rising CVD-risk, particularly above six to seven hours sedentary time per day^11,59^. Importantly, whilst WE group was very close to this threshold in the current study (approx. 6.5 h sitting/day), the SAs exceeded it (approx. 7.8 h sitting/day), suggesting that sedentary behaviour is likely to contribute to increased CVD risk in both populations but to a greater extent in young adult SAs.

Overall, our findings align with existing evidence in adult populations^17,18^, and children^60,61^ that young adult SAs are less physically active than WEs and suggest that a lifetime of inactivity is likely to exaggerate their risk of CVD^10,13,54^. Although the mechanisms by which prolonged sedentary behaviour may contribute to increased CVD risk are poorly understood, there is evidence to that endothelial dysfunction plays a role, along with increased oxidative stress, decreased nitric oxide bioavailability, increased inflammation and elevated levels of circulating adhesion molecules^56^.

Overall this strongly suggests that young adult SAs would benefit from becoming more physically active in order to improve their future CV health. Unfortunately, SA culture does not traditionally encourage exercise from childhood, particularly in young girls, and it has been proposed that a lack of understanding amongst SAs on the health benefits of PA contributes to their low activity levels. Common barriers to participation include concerns about personal safety, physical modesty, lack of confidence, family commitments, and lack of time due to long working hours, whilst participation is often motivated more by social and recreational opportunities than health benefits^62^. Therefore, initiatives to promote activity should focus on encouraging group-based activities, since strong communities, religious and family ties often make it difficult for SAs to participate in physical activity as individuals. Visible role models, both well-known sports figures and local community members, could also play a key role in inspiring participation in physical activity, whilst women-only facilities and encouraging accessible options, such as walking through organised programmes and safe trails, may help to overcome documented barriers^63^.

### Dietary habits

Habitual dietary intake was assessed by three-day food diaries, we well as by FFQ, which captures dietary intake during the previous year. We found good correlations between daily nutrient intake assessed by the two techniques amongst both WEs and SAs. This supports the credibility of both methods and the mean Pearson’s r coefficient of 0.629 for all nutrients was above the reported value of 0.4 which confers validity of techniques used^64^. It is important to note that, whilst the FFQ represents habitual diet across a year-long period, the food diary only represents diet in a very limited time period which may not be representative of their year-long dietary habits and hence a perfect correlation cannot be expected^33^. The mean ratio of energy intake to expenditure calculated from the food diary was within the ‘acceptable’ range of 0.76–1.24, whereas for the FFQ this was slightly lower, suggesting that this technique may lead to ‘under-reporting’ of energy intake. Nonetheless, since the FFQ and food diaries provide unique outlooks on dietary analysis, their use in combination within this study allows for a broad understanding of ethnic differences in diet.

Both the FFQ and food diaries show that young WEs consume significantly more sodium than SAs. Previous evidence has also shown greater salt consumption amongst Scottish women, compared to SA immigrants, in a study of middle-aged women^65^. The findings of the present study show there is already approximately a 1 g difference in young WEs compared to SAs, a level considered to confer up to a 6% increase in CVD risk with increasing sodium consumption^66^. Indeed, according to the present study, only SA women consume less than the guideline *maximum* sodium intake of 2000 mg per day^66^, suggesting that in both WEs and SA men, sodium intake may elevate their CVD risk.

Furthermore, the food diary identified ethnic differences in intake of various nutrients, including sugar, fat, saturated fat, fibre, sodium, vitamin C and folate, with WEs having higher consumption of all of these nutrients compared to SAs. Previous literature from food diaries ranging from four- to seven- days duration supports ethnic differences in nutrient intake^65,67^, and several 24 h dietary recall studies also highlight differences in nutrient consumption in groups of older WE and SA adults^68^. Our study shows for the first time that some of these ethnic differences in dietary patterns are already present in young adulthood.

Importantly, many of the nutrients for which ethnic differences were observed in the present study have been implicated in CVD risk^26^. For example, both WE and SA men and women fall ~ 10 g short of the recommended daily fibre intakes of 38 g for men and 25 g for women^69^. This suggests that both WEs and SAs would benefit from increasing their fibre intake; a 10 g increase in fibre intake has been shown to confer a 9% reduction in CV-mortality^70^.

Furthermore, flavonoids are proposed to have cardioprotective benefits, by reducing blood pressure and improving endothelial function^71,72^. Whilst the exact mechanisms by which this occurs are unclear, increasing flavonoid intake has been suggested to contribute to enhanced bioavailability of endothelial-derived nitric oxide, decreased superoxide-mediated nitric oxide breakdown, improvement in serum lipids and improved inflammatory status^73^. In both ethnic groups, total flavonoid intake in young adult SA and WE men and women as assessed by both the FFQ and food diary was lower than the previously estimated global consumption of 400 mg per day^31^, although mean global intake values are thought to vary between 150 and 600 mg/day^29^.

Total flavonoid intake estimated from the food diary in the present study was ~ 179 mg/day and ~ 76 mg/day for WEs and SAs respectively; the ~ 100 mg difference between these is thought to confer a 7% reduction in CVD risk^30^ and 4% reduction in CV-mortality with increasing flavonoid intake^74^. There is mounting evidence from epidemiological studies of a dose-response relationship between flavonoid intake and reduced CVD risk, which is proposed to plateau around 250–500 mg^75,76^. Both WE and SA consumption was below this level, suggesting that both groups would benefit from increasing their daily flavonoid intake. It has also been proposed that flavonoid-associated CVD benefits may be greater in populations with higher CVD-risk^76,77^, therefore increasing flavonoid intake may be particularly beneficial for young SA men and women.

Overall, the focus of the present study on a young adult population within a narrower age range enables more reliable ethnic comparisons to be drawn without the confounding effects of ageing per se and the changes in diet that might occur with age, as in many other studies. This is particularly relevant for SAs as generational differences and length of residence in Western countries has been shown to influence their dietary practices^68^. The present study showed that young SA adults consume smaller quantities of nutrients that are shown to be beneficial towards cardiovascular health (for example fibre, vitamin C, folate or flavonoids) but they also consume less of the nutrients that may contribute to increased CVD risk (such as saturated fat, sugar, alcohol and sodium) compared to young WE adults. There is evidence suggesting that increasing intake of cardioprotective nutrients has a greater impact than consuming less of nutrients that may contribute to increased CVD risk^78,79^, hence the potential contribution of diet to elevated CVD-risk in SAs is likely due to their low consumption of such protective nutrients.

Communal eating and family dining are key traditions within SA culture, with meals typically prepared for the whole family and food choices influenced by all family members. This can make it difficult for individuals to implement dietary changes, highlighting the need for culturally appropriate dietary advice targeting the entire family. Public health campaigns would be useful to educate the community on the importance of consuming foods beneficial to cardiovascular health; for example, school programmes would help to generate impact from a young age, alongside community workshops to facilitate change across the population. These could include recommendations for recipes and substitutions to be used in traditional meals so that SAs can still enjoy family dining traditions whilst implementing healthy dietary changes.

### Gender differences: physical activity and diet

A greater proportion of males were smokers within both WE and SA populations, aligning with previous evidence in older adults^80^. There was no gender difference in physical activity, other than men having a greater higher level of vigorous intensity activity than women. This is in contrast with other studies, which include wider and older range groups, suggesting that men are more physically active than women^81,82^. Men consumed more alcohol than women, as has been previously reported in a systematic review including studies across broad age ranges^83^. Across both habitual diet and food diary, men had higher intake than women for many nutrients including carbohydrates, fat, sugar, protein and sodium; this is consistent with existing literature, and with physiological differences contributing to gender differences in energy requirement^84^. Overall, the findings of the present study in young adults generally align with previous findings in studies of older and wider age ranges, highlighting that many of these lifestyle patterns begin early in adulthood.

### Limitations

Firstly, all dietary measures are self-reported, which may result in under- or over-estimation of portions and nutrient intake^85^. Although the food dairy asked participants to provide specific quantities, scales were not provided and therefore measures were reliant on participants providing their own equipment and the accuracy of their measurements cannot be guaranteed^86^. Furthermore, PA measures were also self-reported. Studies have highlighted that use of accelerometers to monitor activity levels can lead to more accurate representation of PA, as self-reported measures such as IPAQ tended to over-estimate active minutes and under-estimate sitting time^49,87^. It has even been suggested that differences in PA between WEs and SAs may be less pronounced when measured objectively using accelerometers compared to self-reported^88^. As such, the use of more objective measures such as accelerometers would benefit future studies.

The use of FFQs to compare dietary intake between ethnic groups is also limited by the fact that FFQs, such as that used in the present study, utilise a closed list of foods which is typically designed for Western diets and therefore may not accurately capture the diversity of the SA diet^89,90^. This is exemplified by the ratio of energy intake to expenditure being within the ‘acceptable’ range for WEs, but SAs being slightly below this. Future studies could utilise questionnaires specifically validated for the SA diet, such as those used in previous studies^91,92^. The estimation of flavonoid intake by both dietary assessment tools is limited by the reliability of the food composition database upon which it is based, leading to large variability in intake estimates due to assumptions being made where the identical food option was not included^93^. This is particularly relevant for the FFQ, in which questions incorporate groups of food items and estimates of average weight, hence within some categories there will be a wide range of flavonoid content. Furthermore, the food composition database used for analysis of both assessment tools is not specific to the dietary habits of SAs, which is likely to exacerbate the challenges of accurately assessing their flavonoid intake^93^. This highlights the need for FFQ and flavonoid databases better tailored to the SA diet, as well as a flavonoid-specific FFQ, to enable more accurate quantification of flavonoid intake in the present study.

Finally, the timeframe during which the study was conducted spans the COVID-19 pandemic. This was shown to have altered diet, both negatively and positively, in a variety of populations^94^. This may impact interpretation of absolute values, however, given that all participants were UK residents and subject to the same restrictions, any effects of the pandemic would be expected to have affected both groups to the same extent, hence ethnic differences would likely remain consistent.

## Conclusions

Overall, the findings of the present study suggest that the lifestyle choices of young healthy adult SAs, particularly in regard to reduced PA, increased sitting time and reduced intake of healthy foods and nutrients (such as fibre, folate, flavonoids) do contribute to the elevated CVD risk in the SA population. Thus, key target areas for young WE adults, and to a greater extent young SAs, to reduce their CVD risk include reducing daily sitting time by 1–2 h and increasing intake of foods rich in cardioprotective nutrients such as fibre (beans and pulses) and flavonoids (e.g. unprocessed cocoa, green/black tea, berries, grapes).

## Electronic supplementary material

Below is the link to the electronic supplementary material.

Supplementary Material 1

## Data Availability

All data generated or analysed during this study are available from the corresponding author on reasonable request.

## Abbreviations

ANOVA: Analysis of variance
BMI: Body mass index
CVD: Cardiovascular disease
FFQ: Food frequency questionnaire
IPAQ: International physical activity questionnaire
MET: Metabolic equivalent of activity
PA: Physical activity
SA: South Asian
SD: Standard deviation
WE: White European
