# Supplementary material for: Dietary patterns and physical activity in young South Asians and white Europeans and their potential implications for cardiovascular risk
Source: Sci Rep. 2025 Apr 15;15:12969. doi: 10.1038/s41598-025-97605-z (PMC12000287; doi:10.1038/s41598-025-97605-z)
Supplement: Supplementary file 1 — Supplementary Material 1 [file 41598_2025_97605_MOESM1_ESM.docx]

**
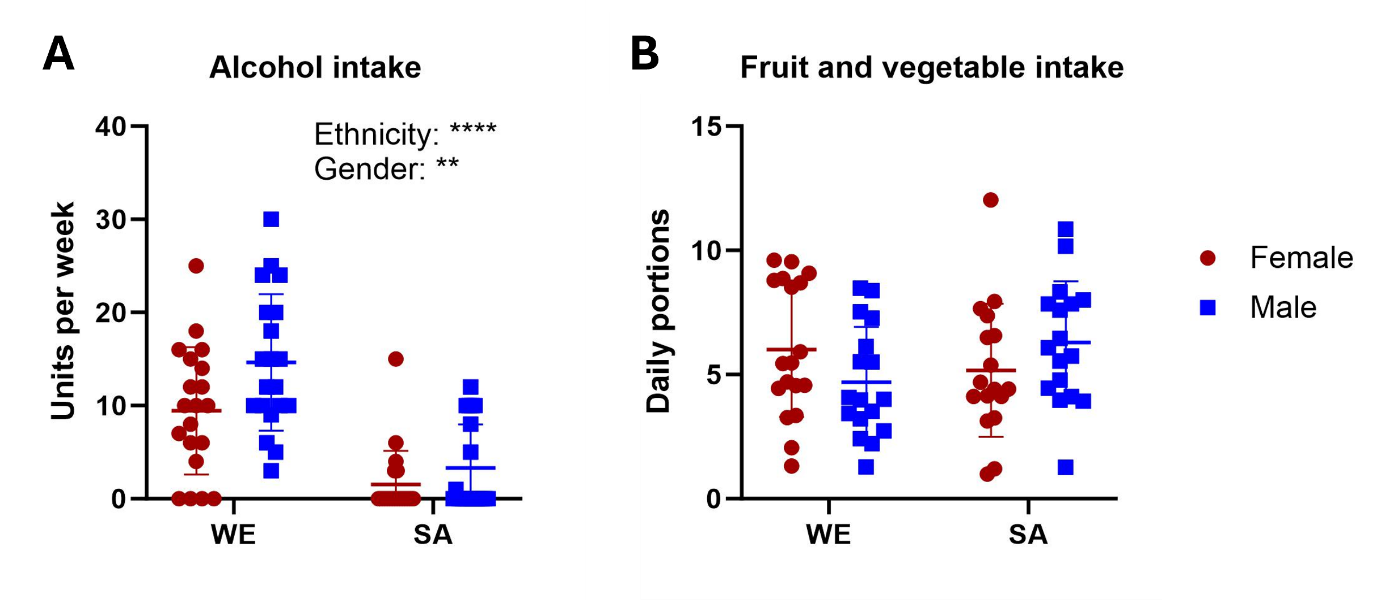
**

**Supplementary Figure 1: Weekly units of alcohol (A) and daily portions of fruit and vegetables (B) for White European (WE) and South Asian (SA) women (red) and men (blue),** as derived from the food frequency questionnaire. Data shown are mean±SD alongside main effects of gender and/or ethnicity are presented from 2-way ANOVA (**: p<0.01, ****: P<0.0001).
